# A Novel Helper qPCR Assay for the Detection of miRNA Using Target/Helper Template for Primer Formation

**DOI:** 10.1155/2022/6918054

**Published:** 2022-04-16

**Authors:** Xianfeng Jiang, Jiahui Wang, Xudan Shen, Jadera Talap, Yang Yang, Dan Yang, Guizhou Xiao, Sheng Cai

**Affiliations:** ^1^Sir Run Run Shaw Hospital, School of Medicine, Zhejiang University, Hangzhou, Zhejiang 310020, China; ^2^Zhejiang Province Key Laboratory of Anti-Cancer Drug Research, College of Pharmaceutical Science, Zhejiang University, Hangzhou, Zhejiang 310058, China

## Abstract

A novel, simple, and sensitive quantitative polymerase chain reaction (qPCR) technology, which is termed as helper qPCR, was established to detect miRNA. In this assay, the target miRNA sequence was introduced as helper template for a reaction switch preforming two-step real-time qPCR strategy. Firstly, the reverse primer was reverse transcribed to form “mediator primer” after binding to the target miRNA. Then, the mediator primer was further extended to form “active template” with annealing to the mediator template. In the end, the active template was amplified and detected by the qPCR reaction system with the help of reverse and forward primers. The SYBR Green dye was used for fluorescence quantification, which is quicker and cheaper than the fluorescent probes, as the detection limit of this assay was 1 pM. This helper qPCR system can be used for different miRNAs detection by redesigning reverse primer for target, indicating this strategy could afford good performance in detecting multiple miRNAs and has a promising application prospect.

## 1. Introduction

MicroRNAs (miRNAs) are a class of small and endogenous RNAs about 18–25 nucleotides in length. They play a key role in cell proliferation, migration, and apoptosis by negatively regulating the gene expression at the posttranscriptional level, which finally influence a wide range of biological processes [1, 2]. Increasing clinical evidences have found that aberrance of miRNA expression is implicated in the occurrence of cancer, cardiovascular, psychiatric, neurodegenerative, and inflammatory diseases [3]. Due to their stability in biofluids, miRNAs are suggested as minimally invasive diagnostic and prognostic biomarkers for cancers [4–8]. Therefore, the detection of miRNAs is extremely important in its functional research. However, the detection of miRNAs is challenging due to great differences in the expression level of miRNA in different tissues or organs and their highly homologous [9–12].

Traditional techniques have been used previously to detect miRNA including northern blotting [13, 14] and microarray technology [15, 16]. Unfortunately, these methods have their own limitations, such as low detection efficiency and long time-consuming for the northern blotting. In addition, northern blotting needs radioactive labeling, which will cause pollution [17, 18]. Microarray technology has its advantage in high throughput and low sample requirement, but the sensitivity and specificity of this method is relatively limited in detecting miRNA since the synthetic probe is fixed on hydrophobic plastic [19]. During the past decades, the development of nanoprobe, electrochemistry, and isothermal amplification detection methods has improved the sensitivity and specificity of miRNA detection [20–23], but these methods need complex primers and complicated operation. In light of the limitations, it is necessary to develop low-cost and effective methods.

Real-time quantitative polymerase chain reaction (qPCR) is another traditional technology which exhibits exponential amplification [24–28]. For instance, stem-loop real-time qPCR is a cost-effective two-step real-time qPCR for miRNA detection [29]. This technique has characteristics of high sensitivity, broad application and greater precision, which is advantageous for the detection of miRNA compared to other traditional methods. Thus, it has been regarded as a gold standard in miRNA detection technology. In this method, target miRNA was first reverse transcribed to cDNA; then, the qPCR was performed to further amplify the cDNA products. However, for some miRNAs, the primer design is relatively intricate and the quantification may be interfered by false positive results. Furthermore, in order to obtain meaningful and repeatable results, it is necessary to control the integrity of the extracted RNA, investigate and optimize the primer design, cDNA synthesis, and other factors simultaneously [30]. These requirements partly limit its application. To achieve the current requirement of the clinical and point-of-care testing (POCT), it is of great practical significance to develop a kind of simple, rapid, sensitive, and specific nucleic acid amplification technologies which have a promising application prospect. Therefore, it needs to improve qPCR method to be used more widely.

A novel platinum (Pt) detection method with helper qPCR technique was previously established by our research group [31]. Compared with conventional qPCR, helper qPCR system contains a helper template, which could be transferred to the active template for performing qPCR. *G* bases in the helper template could be coordinated by platinum ions to form *G*-Pt-*G* chelate [32, 33], obtaining a platinum-responsive on/off switch. Due to the helper template, a slight change in platinum concentration would significantly change the signal in the qPCR, which would greatly improve the sensitivity of the biological detection. Herein, we introduced this simple and sensitive helper qPCR system for miRNA detection. Target miRNA sequence is introduced as the helper template, which is the reaction switch and reverse transcribed with the reverse primer to form “mediator primer.” The mediator primer binds to the 3′-end of the mediator template, generating an active template by DNA extension. The forward and reverse primers are used to amplify the active template for generating signals by qPCR. Thus, the presence of target miRNA with capacity of binding to the reverse primer will lead to the increase of active template. The absence of target miRNA will inhibit the formation of active template. Therefore, a slight difference in the concentration of miRNA will produce a significant change in the signal. Moreover, in this method, the SYBR Green dye was used for fluorescence quantification, which is quick and cheaper than the fluorescent probes, as the detection limit of this assay was 1 pM. The helper qPCR system can detect different miRNAs by designing reverse primer according to the sequence of target miRNAs, indicating this strategy could afford good performance to detect multiple miRNAs. Hence, this novel qPCR system is simple, time-saving, sensitive, and easy to operate for miRNA detection.

## 2. Experimental

### 2.1. Material and Apparatus

The real-time qPCR was proceeded on a StepOnePlus real-time PCR system (Life Technologies, Inc., Carlsbad, CA, USA). All chemicals were of analytical grade and were used as received. AMV reverse transcriptase was purchased from New England Biolabs. RNAsimple Total RNA Kit was obtained from Tiangen Biotech (Beijing) Co., Ltd., (Beijing, China). Diethyl pyrocarbonate was purchased from Aldrich. The real-time PCR kit reagent (SYBR Premix Ex Taq) was obtained from Takara Biomedical Technology (Beijing) Co., Ltd. (Beijing, China). Other chemical reagents were purchased from Sinopharm Chemical Reagent Co., Ltd., and the oligonucleotides were acquired from Invitrogen Biotechnology Co., Ltd. (Shanghai, China) with the sequences shown in Table 1.

### 2.2. Helper qPCR Assay Procedures

In a typical experiment, two steps were involved: miRNA-reverse primer process and qPCR. For miRNA-reverse primer process, reactions were prepared with AMV reverse transcriptase, reverse primer, 1x AMV buffer, and various concentrations of miRNA (miR-152) for 1 hour at 42°C. Then, RT-PCR was performed in a total reaction volume of 10 *μ*l containing 5 *μ*L 2× SYBR® Premix Ex TaqTM, 0.2 *μ*L ROX (50x), 1 *μ*L Primer mix and Mediator template, 3.8 *μ*L AMV reverse transcriptional product. The qPCR procedure was as followed: predenaturation, 95°C, 30 s; 1–40 cycles; 95°C, 5 s; 60°C, 30 s.

### 2.3. Detection of miRNA in Serum

Serum samples were obtained from the papillary thyroid carcinoma patients in Sir Run Run Shaw Hospital (College of Medicine, Zhejiang University) with ethical approval. The serum was firstly diluted to 5% with reaction buffer. Ct value was used for the quantitative calculation of the helper qPCR reaction. The concentration of miRNA was analyzed with the proposed helper qPCR method.

## 3. Results and Discussion

### 3.1. Design Principle of the Helper qPCR System

**Scheme 1:**
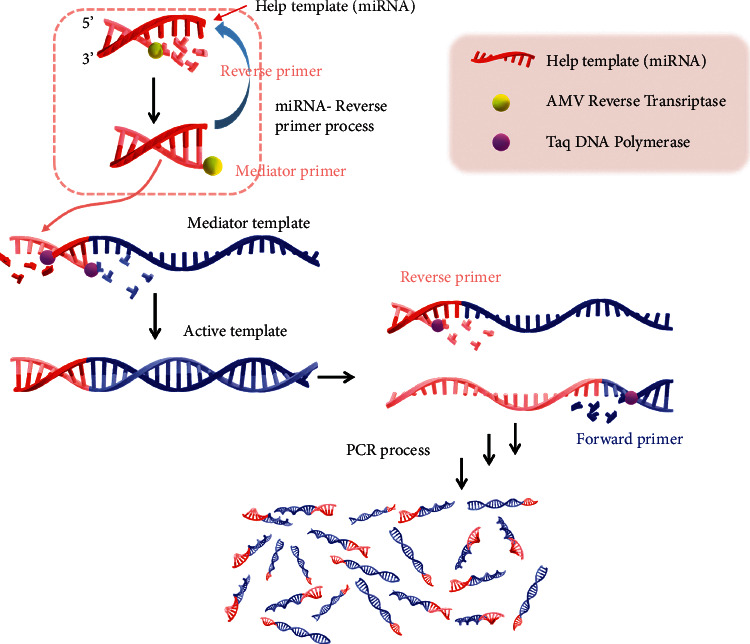
Schematic representation of helper qPCR method for miRNA detection.

In this helper qPCR system for miRNA detection, target miRNA was designed as the helper template. The principle is shown in Scheme 1. The sequence of the reverse primer was complementary to the 3ʹ-end region of the helper template (target miRNA), and the sequence of 5′-end region of the helper template was the same as that of the 3′-end of the “mediator template.” In the presence of target miRNA, reverse primer was combined to the helper template, generating “mediator primer” with the help of AMV reverse transcriptase. Then, the mediator primer was annealed to the “mediator template” and was extended to form an active template for the subsequent qPCR reaction. In the presence of reverse primer and forward primer, the active template was amplified to generate PCR products, binding to which the SYBR Green dye could produce fluorescence signal. The amount of mediator primer was controlled by the amount of miRNA and the efficiency of reverse transcription, thus affecting the qPCR amplification process. Through the design of reverse primer according to the sequence of target miRNA, the helper qPCR system can detect different miRNAs. Therefore, this method is simple, fast, and easy to operate for miRNA detection.

### 3.2. Design of the Reverse Primer

**Figure 1:**
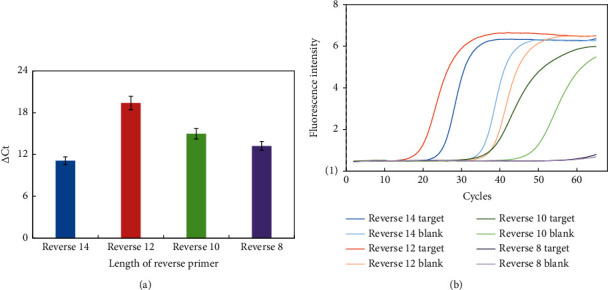
The △Ct value (a) and fluorescence intensities (b) vs different lengths of reverse primer. Experiment conditions: miRNA was 1 nM; forward primer was 4 *μ*M; reverse primer was 2 *μ*M; and mediator template was 50 nM; Tm was 42°C. The detection procedure was carried out as described in the experiment section.

After binding to the reverse primer, successful transcription of the target miRNA is a key point to the detection system. The length of reverse primer will affect the annealing temperature of PCR step and the successful annealing to the template. Therefore, we designed and verified the length of reverse primer. 8, 10, 12, and 14 base number was tried for the reverse primer, and the △Ct value of each primer was investigated under the same reaction condition. As shown in Figure 1, with the increase of reverse primer length, △Ct value increased and reached a maximum (12 bases), then decreased. Thus, reverse primer with 12 bases was selected as the best primer to use for the subsequent experiments.

### 3.3. Optimization of Reaction Conditions

**Figure 2:**
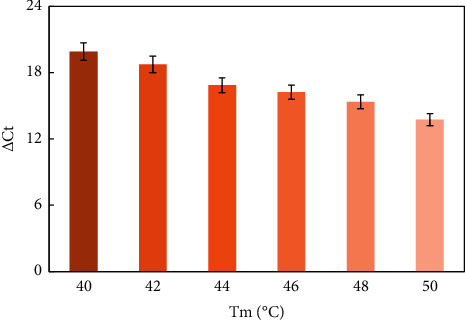
△Ct value vs the Tm. Experiment conditions: miRNA was 1 nM; forward primer was 4 *μ*M; reverse primer was 2 *μ*M; and mediator template was 50 nM. The detection procedure was carried out as described in the experiment section.

**Figure 3:**
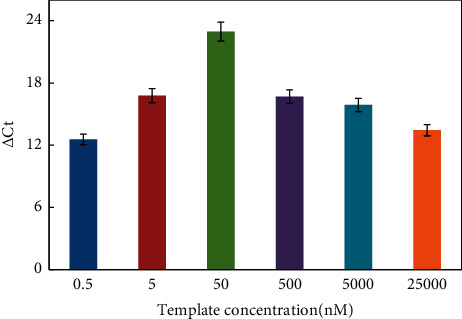
△Ct value vs the amount of template concentration. Experiment conditions: miRNA was 1 nM; forward primer was 4 *μ*M; reverse primer was 2 *μ*M; and Tm was 42°C. The detection procedure was carried out as described in the experiment section.

**Figure 4:**
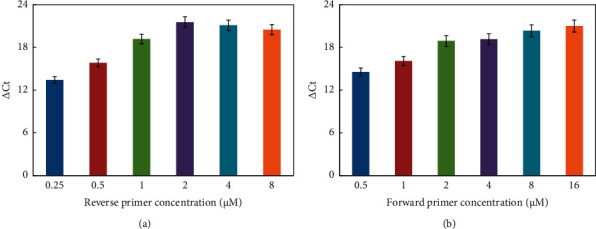
△Ct value vs the amount of reverse (a) and forward (b) primer concentration. Experiment conditions: (a) miRNA was 1 nM; forward primer was 4 *μ*M; mediator template was 50 nM; and Tm was 42°C; (b) miRNA was 1 nM; reverse primer was 2 *μ*M; mediator template was 50 nM; and Tm was 42°C. The detection procedure was carried out as described in the experiment section.

The reaction conditions for the helper qPCR system were optimized by examining the melting temperature (Tm), the amounts of mediator template, reverse primer, and forward primer.  Figure 2 shows that △Ct value was relatively high at low melting temperature, and due to the comprehensive account of blank signal, the temperature of 42°C was chosen as the annealing and extension temperature for the helper qPCR system. The △Ct value increased in the range of 0.5 to 50 nM of mediator template and then apparently decreased (Figure 3). Thus, 50 nM of mediator template was used for further studies. According to Figure 4(a), increasing the amount of reverse primer increased the △Ct value, which reached the maximum at 2 *μ*M and then decreased. Hence, subsequent work employed 2 *μ*M reverse primer. As shown in Figure 4(b), the △Ct value increased with the increasing amount of the forward primer in the range of 0.5 to 4 *μ*M, then, it came into slow growth. Therefore, 4 *μ*M forward primer was selected for further experiments.

### 3.4. Selectivity

**Figure 5:**
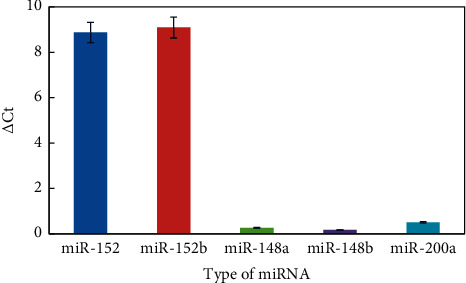
△Ct value vs different type of miRNA. Experiment conditions: miRNA was 50 pM; forward primer was 4 *μ*M; reverse primer was 2 *μ*M; mediator template was 50 nM; and Tm was 42°C. The detection procedure was carried out as described in the experiment section.

To investigate the specificity of the helper qPCR method for miRNA detection, miR-148/152 family members including miR-152 and miR-148, and other family members (miR-200a) were tested.  Figure 5 shows that miR-148a, miR-148b, and miR-200a had almost no effect on the Ct value comparing with miR-152 and miR-152b under the same conditions (50 pM). Therefore, this result indicated that the helper qPCR method can be characterized with high selectivity.

### 3.5. Quantification of the Target miRNA

**Figure 6:**
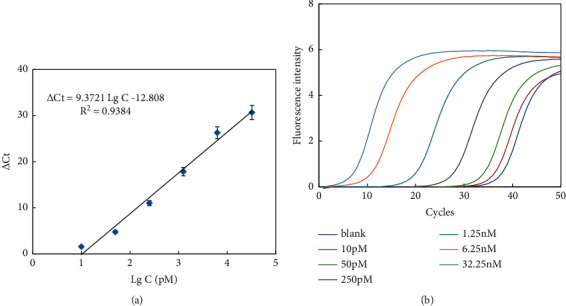
△Ct values (a) and fluorescence intensities (b) for target miR-152 detection. Experiment conditions: forward primer was 4 *μ*M; reverse primer was 2 *μ*M; mediator template was 50 nM; and Tm was 42°C. The detection procedure was carried out as described in the experiment section.

Under the optimized experimental conditions, miR-152 with different concentrations was measured to investigate the ability of the helper qPCR system for quantification of miRNA. As shown in Figure 6, the calibration equation was △Ct = 9.3721 Lg *C*−12.808 with an *R*^2^ (coefficient of determination) of 0.9838 in the range of 10 pM–32.25 nM, where *C* is the concentration of miRNA. Sensitivity testing indicated that this assay can detect less than 1 pM target. The performance of our strategy was equal or better than most previous methods for miRNA detection (Table 2).

### 3.6. Real Sample Detection

To validate the applicability of our method for the miRNA detection in serum sample, a spike experiment was carried out by adding certain amount of miR-152 to the diluted serum, which was used as the biological sample. Different concentrations of miR-152 (10 pM, 250 pM, and 6.25 nM) were successfully determined with the recoveries 117.6 ± 5.56%, 99.7 ± 1.69%, and 108.4 ± 4.86%, respectively (Table 3). This means our method had excellent reproducibility and practicability.

## 4. Conclusions

In summary, we have developed a real-time quantitative detection strategy for miRNA based on helper qPCR system, which has the advantage of simple design, convenient operation, high sensitivity, and selectivity. Compared to conventional real-time qPCR, this helper qPCR system achieved a better performance in miRNA detection due to introducing a helper template. Target miRNA sequences can be used as the helper template for miRNA quantification, achieving high resolution real-time miRNA detection. We expect this simple and feasible method to be a promising technique in biomedical application.

## Figures and Tables

**Table 1:** RNA and DNA sequences used in this work.

Name	Sequence(5′–3′)
miR-152	UCAGUGCAUGACAGAACUUGG
miR-152b	UCAGUGCAUGACAGAACUUGGUU
miR-148a	UCAGUGCACUACAGAACUUUGU
miR-148b	UCAGUGCAUCACAGAACUUUGU
miR-200a	UAACACUGUCUGGUAACGAUGU
miR-152	UCAGUGCAUGACAGAACUUGG
Forward primer	ACCAAGTGGGGCGTTTGGGA
Mediator template R14	ACCAAGTGGGGCGTTTGGGATGTGTTGAGGCCTCCCACAGCCACTGGCCAGCCTGCCTGCTGTCTTCCTGCCCCCCTCGGTCAGTGC
Reverse primer 14	CCAAGTTCTGTCAT
Mediator template (R12)	ACCAAGTGGGGCGTTTGGGATGTGTTGAGGCCTCCCACAGCCACTGGCCAGCCTGCCTGCTGTCTTCCTGCCCCCCTCGGTCAGTGCAT
Reverse primer (12 base)	CCAAGTTCTGTC
Mediator template R10	ACCAAGTGGGGCGTTTGGGATGTGTTGAGGCCTCCCACAGCCACTGGCCAGCCTGCCTGCTGTCTTCCTGCCCCCCTCGGTCAGTGCATGA
Reverse primer 10	CCAAGTTCTG
Mediator template R8	ACCAAGTGGGGCGTTTGGGATGTGTTGAGGCCTCCCACAGCCACTGGCCAGCCTGCCTGCTGTCTTCCTGCCCCCCTCGGTCAGTGCATGACA
Reverse primer 8	CCAAGTTC

**Table 2:** Comparison of sensitivities for different miRNA assay methods.

Method	Amplification strategy	Target	Detection limit
LC-MS	DSN-assisted recycling	miR-21	60 fM [34]
Colorimetric detection	DSN-assisted recycling	Let-7a	1 nM [35]
Fluorescence	Silver nanocluster	Let-7a	14 pM [36]
Fluorescence	T7 exonuclease-assisted recycling	miR-126 and miR-141	15 pM [37]
Fluorescence	RCA and SDA	Let-7a	5 pM [38]
Fluorescence	CHA	miR-21	47 pM [39]
Fluorescence	PCR	miR-152	1 pM (this work)

**Table 3:** Determination of miR-152 in human serum.

Concentration of target added	Concentration obtained with helper qPCR	RSD (%)	Recovery (%)
6.25 nM	6.78 nM	4.86	108.4
250 pM	249.3 pM	1.69	99.7
10 pM	11.8 pM	5.56	117.6

## Data Availability

The data used to support the findings of this study are available from the corresponding author upon request.
